# An unbiased index to quantify participant’s phenotypic contribution to an open-access cohort

**DOI:** 10.1038/srep46148

**Published:** 2017-04-07

**Authors:** Yingleong Chan, Michael Tung, Alexander S. Garruss, Sarah W. Zaranek, Ying Kai Chan, Jeantine E. Lunshof, Alexander W. Zaranek, Madeleine P. Ball, Michael F. Chou, Elaine T. Lim, George M. Church

**Affiliations:** 1 Department of Genetics, Harvard Medical School, Boston, MA 02115, USA; 2 Wyss Institute for Biologically Inspired Engineering, Harvard University, Boston, MA 02115, USA; 3 Program in Medical and Population Genetics, Broad Institute, Cambridge, MA 02142, USA; 4 Program in Bioinformatics and Integrative Genomics, Division of Medical Sciences, Graduate School of Arts and Sciences, Harvard University, Cambridge, MA 02138, USA; 5 Curoverse Inc, Somerville, MA 02144, USA; 6 Department of Genetics, University Medical Centre Groningen, University of Groningen, 9700 RB Groningen, The Netherlands; 7 Personal Genomes.org, Boston, Massachusetts 02115, USA; 8 Division of Genetics and Genomics, Boston Children’s Hospital, Boston, MA 02115, USA

## Abstract

The Personal Genome Project (PGP) is an effort to enroll many participants to create an open-access repository of genome, health and trait data for research. However, PGP participants are not enrolled for studying any specific traits and participants choose the phenotypes to disclose. To measure the extent and willingness and to encourage and guide participants to contribute phenotypes, we developed an algorithm to score and rank the phenotypes and participants of the PGP. The scoring algorithm calculates the participation index (P-index) for every participant, where 0 indicates no reported phenotypes and 100 indicate complete phenotype reporting. We calculated the P-index for all 5,015 participants in the PGP and they ranged from 0 to 96.7. We found that participants mainly have either high scores (P-index > 90, 29.5%) or low scores (P-index < 10, 57.8%). While, there are significantly more males than female participants (1,793 versus 1,271), females tend to have on average higher P-indexes (*P* = 0.015). We also reported the P-indexes of participants based on demographics and states like Missouri and Massachusetts have better P-indexes than states like Utah and Minnesota. The P-index can therefore be used as an unbiased way to measure and rank participant’s phenotypic contribution towards the PGP.

Whole genome genotyping and sequencing technologies have led to several important discoveries in human genetics, and these technologies are now widely used for uncovering the genetic basis in many common and rare human traits and diseases^1^,^2^,^3^. By analyzing genotyping and sequencing data generated from large cohorts of individuals, researchers have discovered the genetic variants associated with a variety of complex human traits and diseases^4^,^5^,^6^. These studies typically start with the recruitment of clinical cohorts of patients with one or more phenotypes of interests. Then, the patients’ DNA samples are collected and genotyped using whole-genome, whole-exome sequencing or whole-genome genotyping using SNP arrays. Some examples of such cohorts include the Framingham Heart Study (FHS) and the Atherosclerosis Risk in Communities (ARIC) Cohort, where patients are recruited primarily for cardiovascular research^7^,^8^. Data from such large cohorts of individuals are conditionally available for other researchers and access can be applied through the database of Genotypes and Phenotypes (dbGaP)^9^,^10^. More recently, projects studies such as the 100,000 Genomes Project aim to perform whole-genome sequencing on a large number of individuals with rare diseases, infectious diseases or cancer^11^.

However, one limitation of these studies with large cohorts of individuals is the ability to re-contact these participants for additional phenotypic data that were not assayed during the study, or for follow-up studies based on the genetic discoveries. This is because most of these studies recruited patients that were not consented to be re-contacted. In a recent large-scale study that looked at 589,306 individuals, the researchers discovered 13 adults with mutations for severe Mendelian diseases and postulated that it will be beneficial to study these 13 resilient individuals for therapeutic purposes. However, these 13 individuals were not allowed to be re-contacted because of the lack of consent^12^.

The Harvard Personal Genome Project (PGP) was developed to overcome such a limitation with studies as described above^13^,^14^,^15^. The PGP invites members of the public to volunteer as participants for genetic research, and aims to perform whole-genome sequencing on every participant. In turn, each participant contributes his or her sequence data along with phenotypic information (such as from their medical records) to the project. To do so, participants can log into their PGP public profile at their own convenience, upload their phenotypic information and perform various survey questionnaires. The data is then made open-access, allowing anyone to use the genotype and phenotype data for research, accelerating the process of using data from large cohorts of individuals for research^16^. These participants have consented the public sharing of their genotype and phenotype data for research purposes, and can be re-contacted for additional follow-up study. Moreover, the PGP does not restrict the inclusion of participants based on a specific medical condition or disease, thus allowing access to a broad repository of individuals with various traits and conditions. However, while participants can choose to participate in the survey questionnaires provided by the PGP and also have the option of providing additional phenotype data, it is unclear how much phenotype data is actually available and how many participants freely participated in providing information for those phenotypes.

In this study, we explored the landscape of phenotypes available in the PGP and how extensive they are using a scoring algorithm, which is unbiased towards any particular phenotype. This algorithm scores both the phenotypes and participants. Briefly, a point is awarded to each phenotype for every unique participant that provided a valid value for that phenotype. For example, if the phenotype is “lung cancer”, valid values include “yes” and “no”. The total number of points for each phenotype is the *Phenotype Score*. The *Phenotype Score* for all valid phenotypes provided by the participants are then added up to calculate the score for each participant. This score is divided by the theoretical maximum score, i.e. the total sum of all valid *Phenotype Scores,* multiplied by 100, which results in a number ranging from 0 to 100. We term this number as the *Participation-index* (or P-index), which gauges the extensiveness in which the participant provided valid phenotypes to the PGP. In determining the P-index, the algorithm allocates more weight to phenotypes that are provided by many participants and gives less weight to phenotypes that are provided by only fewer participants. This is because having more participants with a specific phenotype increases the statistical power for discovering a meaningful genetic association^17^,^18^,^19^. This would encourage participants to prioritize contributing phenotypes that other participants have already contributed. We find that most of the high scoring phenotypes are phenotypes found in the survey questionnaires provided by the PGP. In addition, most participants have either high P-indexes (P-index > 90) or low P-indexes (P-index < 10). We also find that while there are significantly more male than female participants (N_males_ = 1,793, N_females_ = 1,271, *P* = 1.96 × 10^−21^), females on average have higher P-indexes than men (median P-index_male_ = 75.9, median P-index_female_ = 88.1, *P* = 0.015). We also formulated a quantitative trait P-index (QtP-index), which measures the contribution of participants with respect to a list of quantitative traits like height and weight. Finally, we partitioned the participants demographically and reported their P-indexes based on states and zip codes. Thus, using this scoring algorithm, we investigated the landscape of phenotype data available in the PGP, as well as the willingness of participants in providing phenotype data. Our algorithm can be used to incentivize and guide participants (See Discussion) in sharing more phenotype data and can also be applied to other projects similarly structured like the PGP in reaching out to participants for sharing phenotypes.

## Results

### Most high scoring phenotypes were contributed by participants answering survey questionnaires

**Figure 1:**
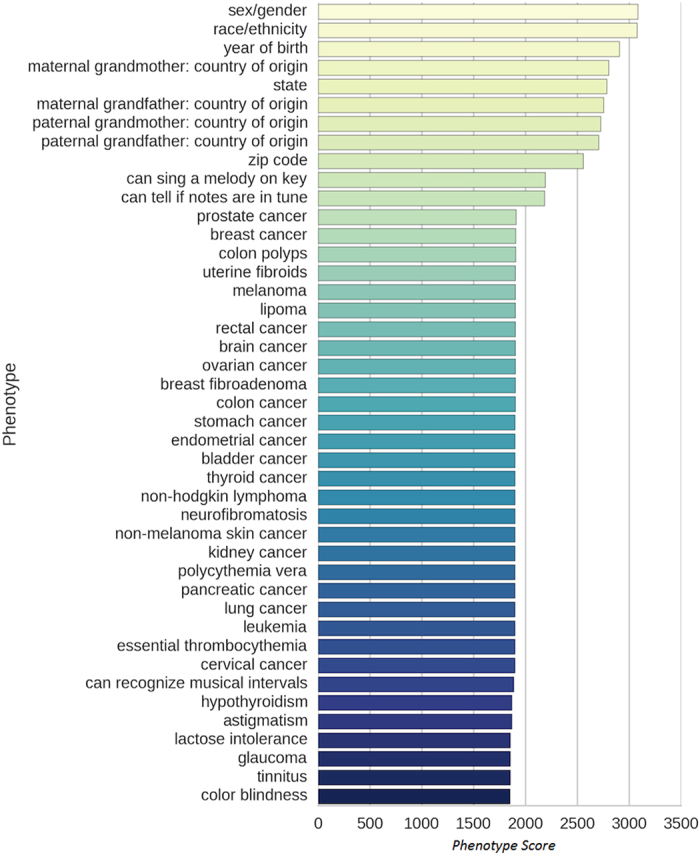
Top 50 phenotypes with the highest *Phenotype Score*. The *Phenotype Score* indicates the number of unique participants with valid values for that phenotype.

We first captured all the participants’ data from the PGP (Table S1), for a total of 5,015 participants and scored each phenotype based on the number of participants who have shared information on the phenotype (see Materials and Methods). Out of the 10,604 unique phenotypes, a total of 2,609 were considered valid based on our filtering criteria (Table S2). Phenotypes were considered valid if 2 or more participants reported valid values for that phenotype, if the phenotype does not pertain to genotyping information and if they meet our other filtering criteria (see Materials and Methods). Out of the valid phenotypes, 262 of these (10.0%) had a score greater than 1,000, indicating that more than 1,000 individuals volunteered information on these phenotypes and 281 (10.8%) had a score greater than 100. The top 50 phenotypes had scores ranging from 1,845 to 3,080 (Fig. 1). Sex/gender was the highest-scoring phenotype, followed by information about ancestry and various diseases, such as breast and colon cancer. All the top 50 phenotypes were directly queried from survey questionnaires. There are 17 different surveys of which 15 are generally available to all participants (Table 1). While most participants provided valid values for these phenotypes by answering the survey questionnaires, there were some participants that provided valid values for them as part of their medical record. The highest scoring phenotype that was not from any survey questionnaires was “diastolic blood pressure” with a score of 250.

### Disease prevalence rates estimated from PGP participants

As most of the phenotypes participants provided were from the survey questionnaires, we proceed to determine the disease incidences for phenotypes found in 10 survey questionnaires, a total of 239 unique phenotypes (Table S3). The disease with the highest prevalence among the PGP participants was “Dental cavities” followed by “Myopia (Nearsightedness)” with more than half the cohort afflicted (79.83% and 53.5% respectively) (Table S4). Other phenotypes with high prevalence include acne, astigmatism, canker sores, urinary tract infection and dandruff (Table S4). Based on our knowledge of some of the diseases listed, some seem to be less common among the PGP participants than the reported population prevalence while some seemed to be higher. For example, the incidence of diabetes mellitus, type 2 in the PGP is 16 out of 1,845 (0.87%) while the reported prevalence ranged from 5% to 14%^20^,^21^,^22^. On the other hand, the incidence of Marfan syndrome in the PGP is 4 (unrelated) individuals out of 1,768 (0.23%) while the reported prevalence ranged from 0.01% to 0.03%^23^.

### Ranking of participants using the *Participation-index* (P-index)

We next calculated the P-index of every participant in the PGP (Table S5). The majority of these participants were of European ancestry, with 2,644 (86.0%) listing their race/ethnicity as “White” (Table S1). The top 10 ranking participants had P-indexes greater than 96.36 (Table 2). The top-ranked participant (hu96713F) had a P-index of 96.735 while the next highest-ranked participants (hu6D1115 and hu199EF4) had P-indexes of 96.716 and 96.603 respectively (Table 2). Given that none of these top-ranking participants have P-indexes of 100, they can provide more phenotypes to improve their P-indexes. For example, hu6D1115 (rank 2^nd^), can provide a valid phenotype for “can recognize musical intervals” (1,843 points), “diastolic blood pressure” (250 points) or “systolic blood pressure” (249 points), either of which would propel her into the 1^st^ rank.

**Figure 2:**
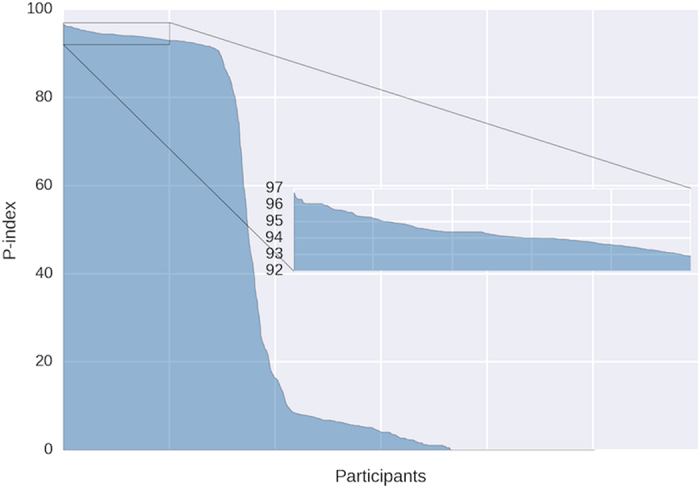
The distribution of the *Participation index* (P-index) for all participants of the PGP, sorted with higher scoring participants on the left and lower scoring participants on the right.

Out of the 5,015 participants, 1,350 participants have a score of 0, indicating that over a quarter of the participants (26.9%) did not provide any valid phenotypes about themselves (Fig. 2). The median P-index is 6.79 and if we filter away the 1,350 participants with no phenotypes, the median P-index goes up to 33.76. The participant scores are mostly dichotomized into 2 distinct groups. Most participants either have P-indexes above 90 (1478 participants) or P-indexes below 10 (2900 participants). These 2 groups of participants make up 87.3% of all participants in the PGP (Fig. 2). As the high-scoring phenotypes are from the survey questionnaires, this implies that most participants either answer most of the surveys or do not take any them, which can explain for the dichotomy of the P-indexes.

### Females tend to share more phenotype data than males

**Figure 3:**
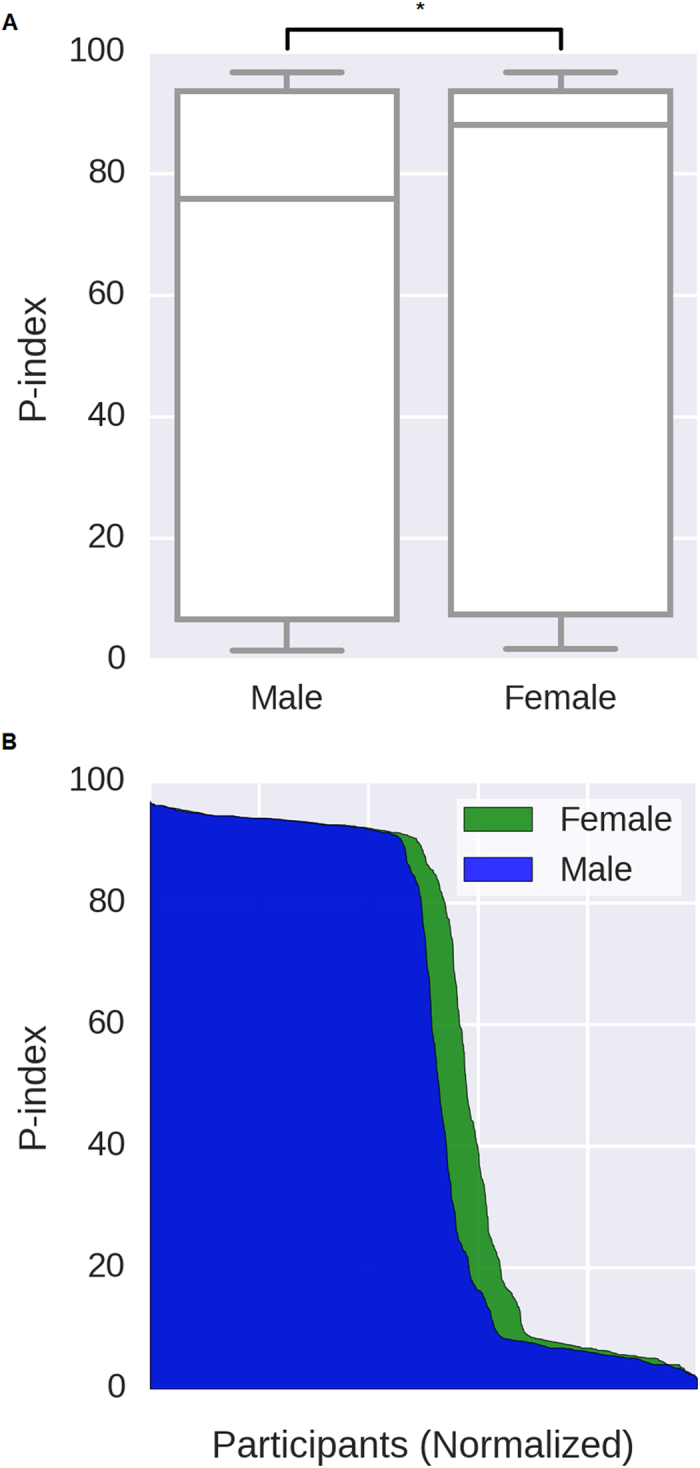
Comparing the P-index of males versus females. (**A**) The P-index boxplots for male and female participants. The female P-index is significantly higher than the male P-index (Wilcox rank-sum, *P* = 0.015). (**B**) The comparison of the P-index distribution between male and female participants normalized by the number of participants for each gender.

Out of the 3,080 individuals who shared their sex/gender, 1,793 of them (58.2%) were males, 1,271 (41.3%) were females and 16 (0.5%) were non-binary or listed both male and female as their gender (Table S6). If we assume under the null hypothesis that males are as likely to participate in the PGP as females, there were significantly more males than females (binomial *P* = 1.96 × 10^−21^). Overall, females have on average higher P-indexes than males (*Wilcoxon rank-sum P* = 0.015, Fig. 3). The median P-index for males is 75.89 while the median P-index for females is 88.09. There were proportionally fewer females with P-index less than 10 (37.0% males versus 31.9% females) and proportionally more females with P-index greater than 90 (46.1% males versus 49.0% females).

### Quantitative trait P-index (QtP-index) to assess and encourage participants to contribute quantitative traits

Some of the phenotypes contributed by PGP participants are quantitative, i.e. they have numerical values. To encourage and guide participants to contribute quantitative traits, we formulated an alternative measure which we call the quantitative trait P-index (QtP-index). Similar to the P-index, we selected 88 quantitative phenotypes (Table S7) and calculated the QtP-index based as well as participant’s ranking based on these 88 phenotypes (Table S8). The top-ranked participant (hu96713F) had a QtP-index of 87.34 and is also the top-ranked participant of the P-index ranking (Table 2). However, none of the other top 10 participants overlapped with the top 10 ranked participants using P-index (Table 2).

### Stratifying the P-index based on geographic location

**Figure 4:**
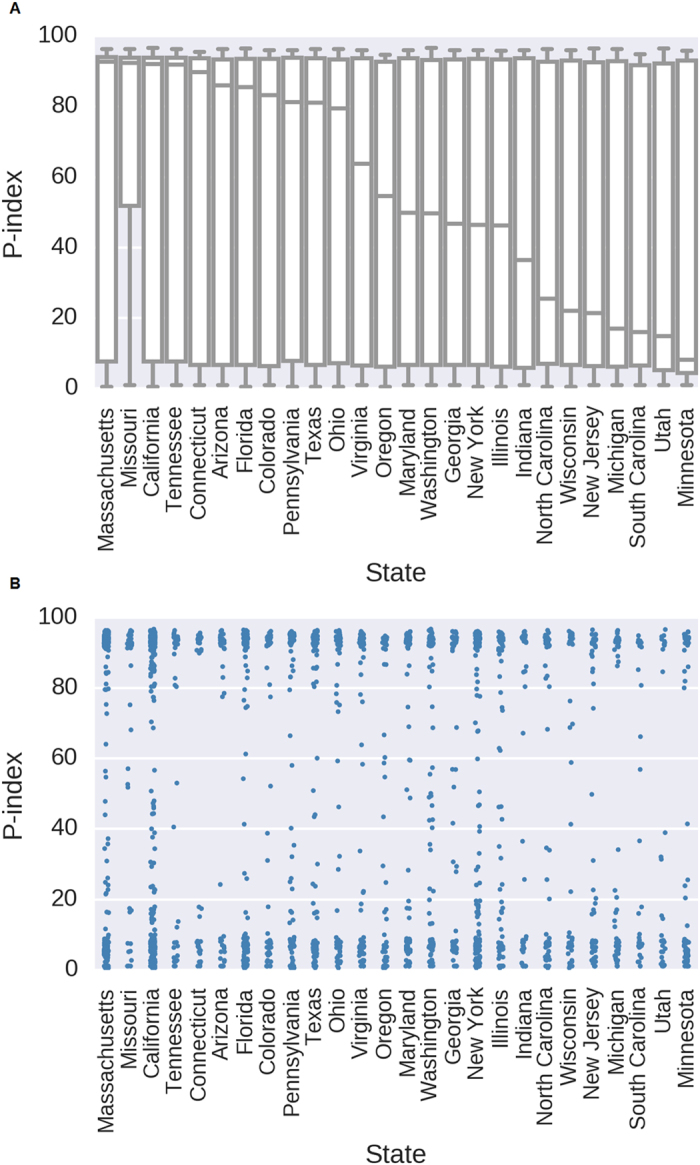
The P-index based on state. Only states with 30 or more participants were included. (**A**) Box-plot of P-index of participants from each state. (**B**) P-index dot-plot of participants from each state.

**Figure 5:**
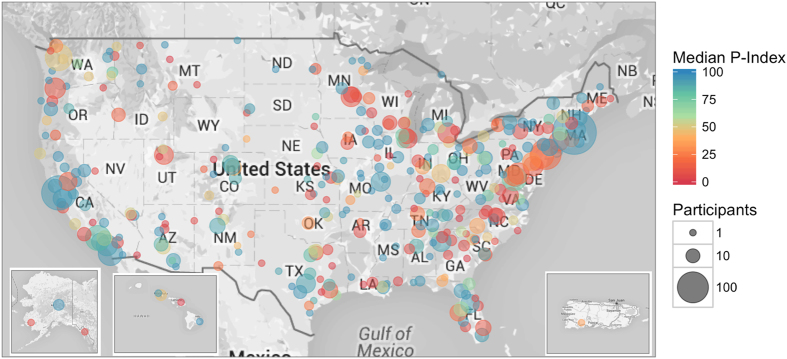
P-indexes by region based on zip codes clustered into 354 distinct groups. The color indicates the median P-index of each group while the size of the circles indicates the number of participants for each group. Figure was generated using R version 3.1.1 (https://www.r-project.org/) and package ‘ggmap’ (Google roadmaps) for the map and package ‘ggplot2’ for plotting^27^,^28^.

We next explored the scores of participants based on geographic location. 2,780 participants reported the states in which they reside in (Table S9). We separated the participants into their respective states and filtered for states that had at least 30 participants, which resulted in 26 states. We then calculated the median P-index as well as the percentage of participants in these states that have P-indexes > 90. Overall, Missouri and Massachusetts performed the best, where the median P-indexes are well over 90 and greater than 60% of the participants has P-indexes > 90 (Fig. 4, Table S10). The state with the lowest median P-index is Minnesota with a median P-index of 8.17. South Carolina has the least number of participants with P-indexes > 90; only 29% of the participants (9 out of 31) had P-indexes > 90 (Fig. 4, Table S10). We also analyzed the P-indexes of participants stratified by zip code. While 2,556 participants provided zip codes (Table S2), only 2,538 were valid to be linked to actual longitude and latitude coordinates (Table S11). We clustered these 2,538 participants into 354 distinct clusters and plotted their median P-indexes (see Materials and Methods). We observed a similar trend as the analysis by state, where the hotspots with participants having high P-indexes are in Massachusetts, Missouri and California (Fig. 5).

## Discussion

The use of a ranking system to rate individuals and institutions is commonly done in many different fields like academia, politics, sports, etc. In academia, we have ranking systems for scientific journals like the impact factor^24^, ranking systems for universities^25^ as well as ranking of individual scientists using the h-index^26^. A common purpose for all ranking systems is act as a performance indicator as well as an incentive for its players to improve their rankings. In this manuscript, we described a method for ranking phenotypes and participants in databases used for research and applied the method to the Harvard Personal Genome Project (PGP). The key feature of the method is that it awards each phenotype a score equal to the number of unique participants that reported a valid value for that phenotype. The rationale for doing so is because the more participants with similar phenotypes, the more statistical power for detecting genetic variants associated with that phenotype^17^,^18^,^19^. Overall, there seems to be an under reporting of potential phenotypes even though there are some high scoring participants. Common anthropometric measurements like height and weight had a phenotypic score of only 1,507 and 1,598 respectively which implies that only a quarter of the participants reported those phenotypes. For the 1,350 participants who had a score of 0, they could bump their P-indexes above 90 by participating in all the available survey questionnaires, which highlights the importance of these survey questionnaires towards obtaining a high P-index.

The algorithm for ranking participants could be adjusted in several ways for measuring the participant’s activity in a more specific manner. A simple adjustment could be to use only a selected number of phenotypes to perform the ranking or to give extra weight to some phenotypes, making the ranking algorithm biased toward theses phenotypes. For example, if one wants to measure or incentivize the participant’s value for cardiovascular research, only phenotypes pertaining to cardiovascular properties like weight, heart rate, cholesterol levels, etc could be used or given more weight in the ranking. In our case, we implemented an alternative P-index called the quantitative P-index (QtP-index) to assess the contribution of participants toward certain quantitative traits. Also, the current algorithm ignores repeated measurement of the same phenotype but one can adjust the algorithm to give extra points to participants for providing multiple measurements of the same phenotypes suitable for the use in longitudinal research studies. Another adjustment could be to have an automated way to combine similar phenotypes together as a single phenotype. For example, in the PGP, the phenotype of “systolic blood pressure” is also sometimes represented as “blood pressure, systolic (upper number)”, “systolic pressure” or “systolic”. By the use of automated text processing, it is possible to reconcile such similar phenotypes to a single phenotype and score the participants with the reconciled phenotype. While these adjustments can improve the ranking system, the current method as described is the first step towards an unbiased and robust ranking mechanism for tracking the phenotypes available and measuring the participation rate of the PGP participants.

There are some other benefits for ranking of participants and phenotypes in databases like the PGP. Besides incentivizing participants to upload as much phenotypes as possible or to determine the possible phenotypes to study, rankings can be used to prioritized participants if the need arises. As participants with rich phenotypes are more useful for research when combined with genotype and biomaterial availability, the rankings can be used to prioritized participants for sequencing and banking of cells. The participant’s ranking could also be used for the purposes of rewarding highly contributing participants by providing them with some reward, e.g. attendance to conferences, consultation about their genomes, etc.

However, there might be potentially some drawbacks in performing the rankings. It could provide an incentive for participants to lie about their phenotypes or to upload phenotypes that have no value and its only purpose is to boost the participant’s ranking (junk phenotypes). However, there are several reasons why this would not be problematic. First, participants are scored not for having the phenotype but for having a valid value or entry for that phenotype. For example, if the phenotype were “Diabetes Mellitus, Type 2”, participants with diabetes will be scored similarly to participants without diabetes if they declared their diabetes status (either yes or no), somewhere in their public profile. Therefore, in this case, lying about the one’s phenotype does not improve your P-index. Next, while participants can upload junk phenotypes in a quest to improve their scores, since only phenotypes with scores greater than 1 will be used, these phenotypes would be irrelevant unless another participant uploads the same phenotype. Thus, cases for such misdemeanor would be rare, as it would require collusion with another participant. Furthermore, the cutoff score could always be adjusted upwards if this indeed becomes a problem.

Also, the PGP is unlike a traditional cohort where phenotypes are contributed by participants online. The argument could be made that the accuracy of the phenotypes contributed, especially for those provided by the survey questionnaires are less accurate than that of a traditional medical cohort. Indeed, for most of our disease surveys, participants are presented with an online form in which they are presented with a list of diseases and conditions and they place a check next to the disease that they are afflicted with. As such, if participants place a check on the wrong box, or fail to place a check on the right box, the surveys will erroneously report wrong values for the phenotypes submitted. There could be ways to mitigate such error like having the system perform error checking or to have the participants reconfirm their phenotype status before submitting the surveys. However, given the open-access, open-consent nature of the PGP, participants can always be re-contacted to confirm their submitted phenotypes.

In summary, we have developed a method to compute the scores of participants and phenotypes in a database for epidemiological and genetic association studies. We applied the method to the Harvard Personal Genome Project (PGP), which returns the score of every phenotype and the P-index of every participant. The phenotype score indicates the number of participants with a valid entry for that phenotype and can be used as a guide for filtering potential phenotypes for research. The participant’s P-index gives an estimate of the overall comprehensiveness of the participant’s phenotypic profile in the PGP and can be also be used to incentivized participants to make their phenotypic profile more comprehensive. Ranking the participants may provide a useful measure with which to compare, in an unbiased way, different participants of the PGP and that ranking can be used as a basis for prioritizing participants for research. Finally, the P-index can be use in a participant-driven cohort like the PGP to study the extent to which participants are willing to share their phenotypic data and evaluate the strengths and weaknesses of such a cohort compared to a traditional population cohort.

## Materials and Methods

### Ethics statement

The Harvard Personal Genome Project (PGP) study is conducted according to the principles expressed in the Declaration of Helsinki. Participation is voluntary and each participant gave informed consent to make their data publicly available. Participants make their data available at their own discretion. All methods presented were carried out in accordance with relevant guidelines and regulations. Experimental and study protocols were approved by Harvard University (Human Research Protection Program).

### Participant recruitment

The Harvard PGP recruit participants via an online sign up page. Participants must pass an online quiz, which they can perform as many times as they want to correctly answer all the questions. The purpose of the quiz is to educate the participant on the benefits and risks of having their genomes, phenotypes and cell lines available publicly. The details of this process can be found on our webpage (http://www.personalgenomes.org/harvard/howitworks) and have been previously described in other publications^14^,^15^.

### Algorithm for scoring phenotypes and participants

The method calculates points for every phenotype and every participant within the database. A phenotype is any data type that any participant uploaded to the database. For example, phenotypes could be “height”, “weight”, “type 2 diabetes” and their values could be “5 feet 9 inches”, “140 lbs” and “yes” respectively. Let *F* be the list of valid phenotypes and *P* is the list of participants. First, the method calculates the score for every unique phenotype. Each unique phenotype is given a score equal to the number of participants with a valid value of that phenotype, i.e.


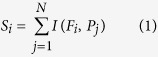


where 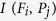 is an indicator function that returns 1 if the participant 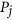 have a valid value for phenotype 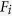 and 0 otherwise. *N* is the total number of participants within the database and *S*_*i*_ is the phenotype score for phenotype *Fi*. The Participation-index (P-index) for each participant is then calculated as the sum of phenotype scores (*S*) that the participant has a valid value divided by the theoretical maximum, i.e.


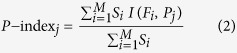


where *M* is the total number of valid phenotypes.

### Downloading and parsing data from the PGP

We implemented a python script to automatically capture all participants’ data from the PGP via its website (https://my.pgp-hms.org/users). We captured the data presented here on July 6^th^, 2016. We iterate through each participant’s profile and extracted every phenotype and its corresponding value. Special characters that encodes for html were replaced by their ASCII equivalent. The resulting phenotypes and their values are stored and formatted in a tab-separated file.

### Survey normalization process

For some of the surveys, only the phenotypes that the participants answered “yes” to are listed on their public profile. For example, for the survey “PGP Trait & Disease Survey 2012: Blood”, there are 13 phenotypes or conditions listed for that survey (Table S3). However, if a participant took the survey but answered no to any of the phenotypes, that information is not listed on the participant’s public profile. As such as part of the normalization process, we reformatted the earlier tab-separated file to fill in the missing phenotypes to indicate that the participants have answered “no” to the phenotypes or conditions listed on the survey (Table S1). We also replaced synonymous phenotypes within the surveys with the same phenotype name. As such, “1.3 --- Weight” was replaced with “Weight”, 1.2 --- Height” was replaced with “Height”, “1.1 --- Blood Type” was replaced with “Blood Type”, “Race” was replaced with “Race/ethnicity”, “Gender” was replaced with “Sex/Gender” and “Date of Birth (mm/dd/yyyy)” was replaced with “Date of Birth”. A list of surveys generally available to PGP participants can be found at https://github.com/PGPHarvard/pgp-surveys/tree/master/Surveys.

### Scoring phenotypes from the PGP

We implemented a script that is calculates the phenotypes scores by implementing the algorithm for scoring as described above. The script takes in the tab-separated file processed earlier (Table S1) and calculates the score for each phenotype by iterating through every individual. The script ignores multiple entries of the same phenotype for each participant and counts them only once. For each participant, the script excludes phenotypes with invalid values. Invalid values are values deemed to be ambiguous and have no potential value for research. These values are “unsure”, “not applicable”, “other/don’t know/no response”, “no response”, “not sure” and “unspecified”.

### Calculating the P-index (scoring participants) from the PGP

The phenotype scores calculated earlier were used as input to another script, which calculates the participant’s P-index using the algorithm described above. We omitted several phenotypes from the P-index calculation that we deem not to be true phenotypes. Phenotypes like “enrolled”, “consent”, “account created”, “eligibility screening”, “participant id” and “exam” have a score of 5,015, indicating that these phenotypes are generated by default for every participant in the PGP regardless of whether they provided any true phenotypes and therefore excluded (Table S2). Phenotypes that pertain to the participant’s genotype were also excluded. These are phenotypes that are deemed to be information about the participant’s genome, i.e. whole-genome sequencing data, whole-exome sequencing data, whole-genome genotyping data, etc (Table S2). On top of that, we also excluded phenotypes with a phenotype score of 1, i.e. the phenotype is not shared by anyone else. This filtering left us with a total of 2,609 valid unique phenotypes that were included for calculating the P-index for every participant (Table S2).

### Calculating the quantitative trait P-index (QtP-index)

The quantitative trait P-index (QtP-index) is calculated in the same way as the P-index but using only a list of phenotypes that have quantitative values. We used only quantitative traits that have a phenotype score greater than or equal to 10, which resulted in 88 such phenotypes (Table S7).

### Calculating median P-indexes by zip code

Out of the 5,015 PGP participants, 2,556 had provided zip code values. The R package ‘zipcode’ was used to clean and filter for valid zip codes that can be linked to latitude and longitude coordinates, resulting in a final dataset of 2,538 participants (Table S9). Participant locations were rounded to the nearest degree by taking the floor of the latitude and ceiling of the longitude and grouped according to their rounded coordinates, resulting in 354 groups. Each group was counted to determine the number of participants and the median P-index. The coordinates used to plot each data point were the median, unrounded latitude and longitude for each group. Map images were obtained using the R package ‘ggmap’ using Google roadmaps and plotted with R package ‘ggplot2’^27^,^28^.

## Additional Information

**How to cite this article:** Chan, Y. *et al*. An unbiased index to quantify participant’s phenotypic contribution to an open-access cohort. *Sci. Rep.* **7**, 46148; doi: 10.1038/srep46148 (2017).

**Publisher's note:** Springer Nature remains neutral with regard to jurisdictional claims in published maps and institutional affiliations.

## Supplementary Material

Supplementary Tables

Supplementary Tables S1-S11

## Figures and Tables

**Table 1:** 

Survey	#Phenotypes	#Participants
PGP10 Trait Survey	23	10
2011 PGP10 CAGI Survey	34	9
PGP Participant Survey	13	2941
PGP Trait & Disease Survey 2012: Blood	13	1794
PGP Trait & Disease Survey 2012: Cancers	25	1898
PGP Trait & Disease Survey 2012: Circulatory System	28	1716
PGP Trait & Disease Survey 2012: Congenital Traits and Anomalies	22	1768
PGP Trait & Disease Survey 2012: Digestive System	23	1724
PGP Trait & Disease Survey 2012: Endocrine, Metabolic, Nutritional and Immunity	18	1839
PGP Trait & Disease Survey 2012: Genitourinary Systems	15	1687
PGP Trait & Disease Survey 2012: Musculoskeletal System and Connective Tissue	23	1724
PGP Trait & Disease Survey 2012: Nervous System	22	1773
PGP Trait & Disease Survey 2012: Respiratory System	9	1697
PGP Trait & Disease Survey 2012: Skin and Subcutaneous Tissue	16	1746
PGP Trait & Disease Survey 2012: Vision and hearing	25	1843
PGP Basic Phenotypes Survey 2015	9	615
Absolute Pitch Survey	4	2583

The various surveys made available to participants along with the number of phenotypes for each survey and number of participants that participated in each survey.

**Table 2:** 

HUID	P-index	Rank	HUID	QtP-index	Rank
hu96713F	96.74	1	hu96713F	87.34	1
hu6D1115	96.72	2	hu016B28	84.03	2
hu199EF4	96.60	3	huF9E138	83.23	3
huC73C2D	96.53	4	huEAA391	82.61	4
hu925B56	96.53	5	hu88A920	82.57	5
hu8BEDE1	96.49	6	hu4C04A0	82.36	6
hu3E50D4	96.42	7	hu7FC65B	82.07	7
hu155D20	96.38	8	huFF6AB4	81.86	8
huF421B2	96.37	9	huD50D1C	81.58	9
huDD6E7A	96.37	10	hu56B3B6	81.45	10

The top 10 ranking participants using P-index (all valid phenotypes) and QtP-index (88 quantitative phenotypes). HUID indicates the PGP ID, which is a unique identifier for every participant of the PGP.
