# Supplementary material for: An unbiased index to quantify participant’s phenotypic contribution to an open-access cohort
Source: Sci Rep. 2017 Apr 7;7:46148. doi: 10.1038/srep46148 (PMC5384003; doi:10.1038/srep46148)
Supplement: Supplementary Tables [file srep46148-s1.pdf]

An unbiased index to quantify participant's phenotypic contribution to an open-access cohort

Yingleong Chan[1,2,3,*], Michael Tung[1,2], Alexander S Garruss[1,2,4], Sarah W Zaranek[5], Ying Kai Chan[1,2], Jeantine E Lunshof[1,3,6], Alexander W Zaranek[5], Madeleine P Ball[7], Michael F Chou[1,2], Elaine T Lim[1,2,8], George M Church[1,2,*]

Affliations

1) Department of Genetics, Harvard Medical School, Boston, MA 02115, USA
2) Wyss Institute for Biologically Inspired Engineering, Harvard University, Boston, MA 02115, USA
3) Program in Medical and Population Genetics, Broad Institute, Cambridge, MA 02142, USA
4) Program in Bioinformatics and Integrative Genomics, Division of Medical Sciences, Graduate School of Arts and Sciences, Harvard University, Cambridge, MA 02138, USA
5) Curoverse Inc, Somerville, MA 02144, USA
6) Department of Genetics, University Medical Centre Groningen, University of Groningen, 9700 RB Groningen, The Netherlands
7) PersonalGenomes.org, Boston, Massachusetts 02115, USA
8) Division of Genetics and Genomics, Boston Children's Hospital, Boston, MA 02115, USA

*Corresponding authors

George M Church (gchurch@genetics.med.harvard.edu)

Yingleong Chan (ychan@genetics.med.harvard.edu)

Supplementary Tables Description

Table S1 – All Data

This table shows a data dump of the Harvard Personal Genome Project (PGP) of every participant (https://my.pgp-hms.org/users) after going through the survey normalization process (See Materials and Methods). This data dump is performed on July 6th, 2016.

HUID – Harvard University Identity, the primary key for every participant of the PGP. They key begins with the letters “hu” followed by 6 alphanumeric characters. This key is unique for every PGP participant.

Fieldname – The field or phenotype recorded on the participant’s public PGP profile.

Value – The value of the above field or phenotype.

Table S2 – Phenotype Score

This table shows the phenotype score of all the phenotypes determined from processing Table S1.

Phenotype - All phenotypes formatted to lower case.

***Phenotype Score* –** The *Phenotype Score*, which is the number of participants with a valid value for that phenotype.

Status – The status indicating whether the phenotype is included in the P-index calculation, which can be “Include” or “Exclude”.

Table S3 – Disease Surveys

This table shows the phenotypes from the 12 disease surveys available to participants. The results on this table aggregate the phenotypes present in the surveys as well as medical record data that participants upload.

Phenotype – The phenotypes listed for each of the survey.

No. of Yes – The number of participants indicating that they are affected by the phenotype.

No. of No – The number of participants indicating that they are unaffected by the phenotype.

% Yes – The percentage of participants affected by the phenotype.

Table S4 – Disease prevalence

Like Table S3, this table shows the phenotypes from the 12 disease surveys available sorted by disease prevalence.

Phenotype – The phenotypes listed for each of the survey.

No. of Yes – The number of participants indicating that they are affected by the phenotype.

No. of No – The number of participants indicating that they are unaffected by the phenotype.

% Yes – The percentage of participants affected by the phenotype.

Table S5 – P-index ranking

The rankings of all participants using the P-index.

HUID – Harvard University Identity (See Table S1).

Points – The total number of points awarded to each participant. Use as a measure to calculate the P-index.

P-index – The index used to rank every participant, which is a normalized score of the number of points for each participant.

Rank – The ranking for each participant ranging from 1 to T3667. The letter "T" preceding the rank indicates 2 or more ties, i.e. 2 or more participants have the same P-index and therefore are tied in ranking.

Table S6 – P-index gender

The P-index of all participants that have indicated their gender.

HUID – Harvard University Identity (See Table S1).

P-Index – The index used to rank every participant (See Table S5).

Gender – The gender/sex of the participant.

Table S7 – QtPhenotypeScore

The phenotype scores for the 88 quantitative traits/phenotypes used for the QtP-index (See Table S8).

Phenotype – The quantitative phenotype

Phenotype Score - The *Phenotype Score* for the quantitative phenotype

Table S8 – QtP-index ranking

The rankings of all participants using the quantitative P-index (QtP-index).

HUID – Harvard University Identity (See Table S1).

Points – The total number of points awarded to each participant from the quantitative trait phenotypes. Use as a measure to calculate the QtP-index.

QtP-index – The index used to rank every participant using the QtP-index.

Rank – The ranking for each participant ranging from 1 to T1642. The letter "T" preceding the rank indicates 2 or more ties, i.e. 2 or more participants have the same QtP-index and therefore are tied in ranking.

Table S9-P-index state

The P-indexes of participants who contributed information about which state within the United States they reside in.

HUID - Harvard University Identity (See Table S1).

P-Index – The index use to rank every participant (See Table S5).

State – The state of residence of that participant.

Table S10-median P-index state

The median P-index of every state where there are at least 30 participants.

State – The state of residence of that participant.

No. of Participants – The number of participants from that state.

Median P-index – The median of all the P-index values of participants from that state.

% P-index > 90 – The percentage of participants with P-index greater than 90 from that state.

Table S11-P-index zip code

The data used for mapping each participant to a more specific location using their submitted postal zip codes.

HUID - The Harvard University Identity (Table S1)

P-index – The index used to rank every participant (See Table S5).

Provided zip code – The zip code provided by participants.

Formatted zip code – The actual zip code used.

City – The city located using the formatted zip code.

State – The state located using the formatted zip code.

Latitude, Longitude – The map coordinates to pinpoint the location of the participant.

Group ID – The local grouping of each participant. Participants in the vicinity are given the same Group ID.
